# Perceptions and current practices of community pharmacists regarding antimicrobial stewardship in Tasmania

**DOI:** 10.1007/s11096-018-0701-1

**Published:** 2018-08-02

**Authors:** Tasneem Rizvi, Angus Thompson, Mackenzie Williams, Syed Tabish Razi Zaidi

**Affiliations:** 1 Department of Pharmacy, School of Medicine, University of Tasmania, Hobart, Australia; 2 School of Healthcare, University of Leeds, Leeds, UK

**Keywords:** Antimicrobial, Australia, Perception, Pharmacist, Practice, Antimicrobial stewardship, Survey

## Abstract

*Background* Despite increasing interest in antimicrobial stewardship (AMS), little is known about the related practices and perceptions of community pharmacists. *Objective* To develop and validate a questionnaire to measure the current practices of, and barriers to community pharmacists’ participation in AMS. *Setting* Community pharmacists in Tasmania, Australia. *Method* A questionnaire to explore AMS knowledge, current practices and perceptions of community pharmacists was developed. It was designed after rigorous literature review, expert opinion, and feedback from a group of community pharmacists. A convenience sample of 140 Tasmanian community pharmacists was used for this study. Cronbach’s alpha and exploratory factor analysis (EFA) were used for reliability and validity. The questionnaire was hosted online, a link to which was sent by invitation e-mails, fax and post to community pharmacists in Tasmania, Australia. *Main outcome measure* Current AMS practices, perceived importance, barriers and facilitators of AMS. *Results* Eighty-five pharmacists responded to the survey yielding a response rate of 61%. EFA identified one factor solution for each of three perceptions scales and showed acceptable reliability. The Cronbach’s alpha of perceived importance-understanding was 0.699, perceived importance-motivating was 0.734, perceived support from GPs was 0.890, operational barriers was 0.585, general facilitators was 0.615. Most pharmacists reported that they counselled patients on adverse effects (86%), drug interactions (94%), and allergies (96%). In contrast, less than half (43%) intervened with prescribers regarding antibiotic selection. Lack of training, lack of access to patients’ records, limited interactions with general practitioners and absence of a reimbursement model were major barriers limiting community pharmacists’ participation in AMS. *Conclusion* The questionnaire was of acceptable reliability and validity; a larger study will further contribute in its reliability and validity. Future studies utilising the questionnaire at national and international level may provide further insights into the determinants of community pharmacist’s involvement in AMS.

**Electronic supplementary material:**

The online version of this article (10.1007/s11096-018-0701-1) contains supplementary material, which is available to authorized users.

## Impact of findings on practice statements

- An improved understanding of routine practices and perceptions of community pharmacists related to antimicrobial stewardship can assist in the development and implementation of antimicrobial stewardship related initiatives in community settings.
- Community pharmacists are willing and capable of playing an important role in helping optimise antimicrobial use by educating patients and effectively interacting with prescribers, though a number of barriers may currently be limiting their participation.
- Knowledge surrounding current practices and perceptions of community pharmacists regarding antimicrobial stewardship is limited. Future research into the barriers to and facilitators of community pharmacists optimising antimicrobial use is required.

## Introduction

Increasing antimicrobial resistance (AMR) is a major global threat to human health [1]. If not tackled urgently AMR will cause 10 million deaths annually by 2050 [2]. Antimicrobial stewardship (AMS) refers to the coordinated interventions designed to measure and improve the appropriate use of antimicrobials by promoting the selection of the optimal antimicrobial drug, dose, duration of therapy, and route of administration [3]. AMS is high on the agenda of global health organisations and currently there is an increasing interest in community based AMS initiatives, as this is where the majority of antibiotic use occurs, much of which is inappropriate [4].

Community pharmacists can play an integral role in AMS programs within community settings for various reasons. Firstly, pharmacists are delivering value added services beyond their traditional dispensing duties [5]. Secondly, they are one of the most frequently seen healthcare professionals and serve as the first point of contact for seasonal viral respiratory tract infections-the most common conditions where inappropriate use of antibiotics has been noted [6]. Thirdly, community pharmacists often liaise between patients and various service providers, and are well positioned to operationalise any AMS framework. Little is known about the current practices of community pharmacists in the developed world. An improved understanding of these issues can assist in the development and implementation of AMS initiatives in community settings. Some of the previous studies were conducted in countries where antibiotic prescribing and dispensing are neither reimbursed nor well regulated thus making the research findings less applicable in developed countries [7, 8].

In Australia, approximately 27 million antibiotic prescriptions are dispensed annually [9] and antimicrobial use is 20% above the average of countries in the Organisation for Economic Cooperation and Development [10]. To address this issue, the Australian government released its first antimicrobial resistance strategy in the year 2015 [11]. The Australian government-funded National Prescribing Service MedicineWise (NPS-MW) is playing an important role to reduce antibiotic use in the community raising awareness through educational initiatives such as the “resistance fighter campaign” and “antibiotic awareness week” [12]. In Australia, strict regulatory restrictions exist and antibiotics cannot be dispensed without a prescription [13]. However, once a prescription is issued, it is generally valid for 1 year and in many cases prescribing software automatically defaults to including a repeat. Furthermore, dispensing software in Australian community pharmacies have no interface with the prescribing software and therefore, pharmacists cannot access a patient’s clinical information or laboratory data [13]. This further limits Australian community pharmacists participation in AMS initiatives. Little is known about the current practices and perceptions of pharmacists working in the community sector.

The primary aim of our study was to develop and validate a questionnaire to measure the perceptions and practices of Australian community pharmacists regarding AMS. A secondary aim was to determine community pharmacist’s awareness of and engagement with NPS-MW’s initiatives designed to reduce AMR. The study was conducted in the Australian state of Tasmania, which has a population of around 520,000 and a geographical area similar to that of the Republic of Ireland. The study findings can help to inform AMS frameworks for community pharmacists in Australia.

## Ethics Approval

This study received approval from Tasmanian Human Risk Ethics Committee (HREC) in April 2016 (H0015673).

## Method

### Survey development

We generated an item pool based on a thorough literature review with key words related to antimicrobial stewardship, community, and pharmacists [7, 11, 14–20]. STRZ reviewed, modified and organised this item pool according to Australian pharmacy practice. A demographic section was also introduced at this stage. The questionnaire was then reviewed by AT and a section on items related to the NPS-MW initiatives was included. Following the initial review, the questionnaire was edited, based on limited piloting on researchers and practising pharmacists working in the Division of Pharmacy at the University of Tasmania. The final questionnaire is available as supplementary material. A Likert-type agreement scale was used for questions around current practices and perceptions.

### Survey deployment

The questionnaire was hosted online using the Lime Survey^®^ portal. A convenience sample of 140 community pharmacists across Tasmania was invited to participate in the study via e-mail, fax and post during the first week of May 2016. Subsequently copies of questionnaire with a standard invitation letter were faxed and posted to pharmacies where there had been no initial response. The invitation letter included a web link to the questionnaire, a mobile-enabled scanning code (QR code) directing participant to the questionnaire site, and a paper survey with a self-addressed reply paid envelope. Two reminders were sent on a fortnightly basis after faxing/posting. Participants were offered the chance to win one of five gift cards selected by a draw conducted at the end of the study.

### Data analysis

Exploratory factor analysis (EFA) was used to examine the internal structure and construct validity of the perception scales. Maximum likelihood technique with the oblique rotation was employed. The items having a rotated factor loading of at least 0.55 or above [21] were retained for each factor. Qualitative feedback from the participants was discussed amongst the investigators where loadings were ambiguous. Cronbach’s alpha was used to determine the reliability of individual factors. Qualitative comments were analysed using a constant comparative approach to identify various themes under the guidance of STRZ without any specific software. Univariate linear regression was employed to identify variables and factors associated with the participants’ scores on the current AMS practices section of the survey. Variables with a *p* value ≤ 0.20 were included in the multivariate linear regression model. All statistical analyses were performed using SPSS version 22 (IBM Inc., Chicago, IL).

## Results

Eighty-five of the 140 community pharmacists responded to the survey yielding a response rate of 61% with the majority of respondents being female (65%) (Table 1). A wide distribution of age and experience was noted among the participants ranging from 23 to 70 years and 1–50 years respectively. Most participants (80%) had an undergraduate pharmacy degree as their highest qualifications.

**Table 1:** Demographics of survey respondents

Categories	Total (%)
Gender (n = 63)	
Female	41 (65%)
Male	22 (35%)
Age (n = 62)	
21–30	10 (16%)
31–40	21 (34%)
41–50	14 (23%)
51 and above	17 (27%)
Experience as community pharmacist (n = 64)	
Less than 10 years	18 (28%)
10–19 years	15 (23%)
20–29 years	13 (20%)
30 years or more	18 (28%)
Education (n = 65)	
Bachelor’s degree in Pharmacy	52 (80%)
Master’s degree in Pharmacy	3 (5%)
Doctorate degree in Pharmacy	3 (5%)
Other	7 (10%)
Location (n = 65)	
Metro	40 (62%)
Rural	25 (38%)

### Validity and reliability of the survey tool

Appendix 1 in “Electronic supplementary material” shows the results of EFA and Appendix 2 in “Electronic supplementary material” shows the results of reliability analysis including total item statistics. The rotated solution for the perceived importance scales showed two factors comprising of perceived understanding of AMS and perceived motivating factors of AMS (Cronbach Alpha 0.699 and 0.734 respectively). The EFA of perceived barriers scale yielded a two-factor solution comprising of perceptions regarding support from GPs and operational barriers (Appendix 1 in “Electronic supplementary material”). The Cronbach’s alpha of the perceived support from GPs and the operational barriers scale was 0.89 and 0.58, respectively. The EFA of perceived facilitators scale yielded one-factor solution. The Cronbach’s alpha of the general facilitators’ scales was 0.615. Items on monetary compensation and public image of pharmacists’ role in AMS were retained because of a strong support from the qualitative feedback on these issues.

### Results of the study

#### Awareness of NPS-MW initiatives

The majority (63%) of pharmacists knew the term ‘antimicrobial stewardship’, although 75% reported an improved understanding after reading the provided definition. Most respondents were aware of the general (80%) and specific (72%) NPS-MW quality initiatives, though less than half (45%) were aware of the resources available to them. Around a quarter of the respondents reported that they are taking more interest (24%) and making more interventions (27%) regarding antibiotic use due to the NPS-MW’s initiatives. Lastly, nearly half of the participants (53%) reported that they would be willing to participate in the future AMS initiatives if resources are made available.

#### Current practices of AMS

Pharmacists frequently contacted prescribers relating to allergies, dosing, or drug interactions (Table 2). On the contrary, pharmacists less commonly contacted prescribers if they considered the choice of antibiotic to be inappropriate.

**Table 2:** Current AMS practices of Tasmanian community pharmacists

Scale and items	Participant’s response, %		Median (IQR)
	Scoring ≤ 3	Scoring ≥ 4	
Current AMS practices			
Providing clear messages on expected side effects (n = 72)	13.9	86.1	4 (4–5)
Providing clear messages what should be done if patient experience side effect (n = 72)	22.2	77.8	4 (4–5)
Contacting the prescriber if the patient is allergic to the prescribed antibiotic (n = 72)	1.4	98.6	5 (5–5)
Contacting the prescriber if the antibiotic dose/frequency is too high or too low (n = 71)	14.1	85.9	5 (4–5)
Contacting the prescriber if the prescribed antibiotic involves a drug interaction (n = 70)	2.9	97.1	5 (5–5)
Contacting the prescriber if the choice of antibiotic may not be optimal (n = 71)	53.5	46.5	3 (2–4)
Recommending OTC/self-care treatment to patients with symptoms of infection not needing antibiotics (n = 71)	4.2	95.8	5 (4–5)
Referring patients to a general practitioner when symptoms are suggestive of an infection (n = 69)	1	99	5 (5–5)
Providing advice when it would be appropriate to use the repeat (n = 70)	17.1	82.9	4 (4–5)
Discussing with patient to determine if it is appropriate for them to use the presented repeat (n = 72)	30.6	69.4	4 (3–5)

Current practices measured on a scale of 1–5, where 1 = do not practice at all and 5 = practice all the time

*n* Number of participants, *IQR* inter quartile range

Respondents indicated that they were referring patients to see GPs if they suspected an infectious presentation that might need an antibiotic prescription, but where this was not the case Pharmacists reported that they were invariably managing patients by offering over the counter medicines (95.8%). Pharmacists were commonly ascertaining the need for an antibiotic when a patient presented a repeat prescription (82.9%).

#### Perceptions and association with AMS practices

Most pharmacists agreed that AMS programs in community pharmacy would lead to a reduction in inappropriate antibiotic use and the costs associated with managing infections (Table 3). Similarly, pharmacists believed that they could play an important role in implementing AMS initiatives and their participation in AMS programs would lead to a better public image and enhanced job satisfaction. Pharmacists also indicated that the lack of access to patients’ medical records and objective laboratory information limited their participation in AMS. Pharmacists also felt that GPs do not welcome their intervention regarding choice of antimicrobial prescription (Median = 5, IQR 5–6.25 Scale 1–7). On the contrary, interventions related to the dose, duration or dosage form of antibiotics were perceived as welcomed by GPs. Pharmacists were mostly neutral about the lack of training as a barrier to their participation in AMS. Likewise, most of them did not consider lack of time as a barrier in their AMS role (Table 3). Facilitators related to public awareness campaigns, collaboration with GPs, access to antibiotic guidelines and patients’ clinical and laboratory data, were all considered as most helpful in increasing pharmacists’ AMS involvement (Table 4).

**Table 3:** Perceived importance and barriers to participate in AMS in community pharmacy

Scales and items	Participant’s response, %		Median (IQR)
	Scoring ≤ 4	Scoring ≥ 5	
Perceived importance of AMS-understanding of the role			
Community pharmacist can play an important role in AMS (n = 68)	2.9	97.1	7 (5–7)
AMS will reduce health care costs associated with infections (n = 68)	21.6	78.4	7 (5–7)
AMS will reduce inappropriate antibiotic use (n = 68)	17.6	82.4	5 (5–7)
Perceived importance of AMS-motivating forces			
AMS will enhance the public image of pharmacists (n = 67)	20.9	79.1	6 (5–7)
AMS will enhance the job satisfaction of pharmacists (n = 67)	17.9	82.1	6 (5–7)
Perceived barriers of AMS-operational barriers			
I do not have the required training to participate in AMS (n = 66)	63.6	36.4	4 (3–5)
I do not have enough time to participate in AMS (n = 64)	75	25	3 (3–5)
Limited access to patient record to review the appropriateness of antibiotic prescriptions (n = 65)	4.6	95.4	6 (5–7)
There aren’t any standard guidelines to implement AMS (n = 62)	33.9	66.1	5 (4–6)
Perceived barriers of AMS-perceived support from GPs			
GPs are not receptive to pharmacists intervening on the choice of antibiotic (n = 63)	34.9	65.1	5 (5–6.25)
GPs are not receptive to pharmacists intervening on the dose and dosage form of antibiotic (n = 64)	64.1	35.9	3 (3–6)
GPs are not receptive to pharmacists intervening on the duration of antibiotic (n = 62)	75.8	24.2	3 (3–6)

Perceived importance and perceived barriers were measured on a scale of 1–7, where 1 = strongly disagree and 7 = strongly agree

*n* Number of participants, *IQR* inter quartile range

**Table 4:** Perceived facilitators of AMS in community pharmacy settings

Scales and items	Participant’s response, %		Median (IQR)
	Scoring ≤ 3	Scoring ≥ 4	
Perceived facilitators of AMS-General facilitators			
Increased provision of education activities regarding AMS (n = 65)	6.2	93.8	5 (4–5)
Better collaboration with local GP practices (n = 65)	1.5	98.5	5 (4–5)
Clarifications of the duties of pharmacists’ professional organizations (n = 63)	27	73	4 (3–5)
Better access to patient’s clinical and laboratory data (n = 64)	7.8	92.2	5 (4–5)
Perceived facilitators of AMS-operational facilitators^a^			
Public awareness initiatives highlighting community pharmacists in AMS (n = 66)	10.6	89.4	5 (4–5)
Monetary compensation for the time involved in AMS programs (n = 64)	18.8	81.2	4 (4–5)

Perceived facilitators measured on a scale of 1–5, where 1 = Unhelpful and 5 = most helpful

*n* Number of participants, *IQR* Inter quartile range

^a^Items not loaded on any factor but retained based on qualitative analysis as participants were very vocal about the issues covered by these items

The univariate linear regression analysis identified three variables that showed some degree of association with the total scores for the AMS practices section of the survey (p value ≤ 0.2). The three variables were willingness to participate in the future AMS initiatives, total scores of general facilitators scale and perceived importance scale. The multivariate linear regression analysis did not identify any of these variables having a significant association with the AMS practices of the community pharmacists (Table 5).

**Table 5:** Multivariate linear regression analysis: predictors of Tasmanian Community Pharmacists' participation in AMS (n = 59)

Predictor	Unstandardised β	Standardised β	*P* value	95% CI range
Willingness to participate in future AMS initiatives	0.13	0.05	0.07	− 0.56–0.82
Total scores on the perceived importance scale	0.53	0.25	0.20	− 0.06–1.12
Total scores on the general facilitators scale	0.46	0.17	0.70	− 0.25–1.18

### Qualitative feedback

Qualitative comments were analysed using a constant comparative approach to identify various themes under the guidance of STRZ. Pharmacists showed great interest in providing qualitative feedback via free text comments. The main themes from the qualitative feedback were contextual limitations of community pharmacists, improper use of repeat prescriptions, need of public awareness, lower than recommended dose of antibiotics in children and impact of AMS on business model of pharmacy. Software was not used for qualitative analysis. Details of these comments are presented as Table 6.

**Table 6:** Qualitative feedback from the Tasmanian Community Pharmacists

Theme	Example statements
Contextual limitations	Unlike hospital setting, implementation of AMS is certainly a challenge in the community. GPs prescribe antibiotics due to the pressure of patients. Are there any ID consultants involved in community AMS? Who is going to give approval and decide the duration?
	Not sufficient information about ailment or patient to make a call about appropriateness of antibiotic
	Until we are provided full history, pathology and diagnosis, very difficult to implement
	It is not always easy to determine what infection is being treated in a patient, as we have not made the diagnosis and if the patient can communicate this appropriately then ensuring the most suitable antibiotic can be difficult as it may be specific to a sputum sample, culture etc. This could be a hurdle in AMS
	I think you cannot have an AMS program in community pharmacies without any prior agreement with the prescribing doctors for those pharmacies, otherwise will cause client confusion, and worsen the relationship with doctors. Also considering that pharmacists lack diagnostics skills, it is the role of the doctor to determining the need for antibiotic and not the pharmacist to question the doctor’s decision
Increase public awareness	I always explain the expected duration whether it is less than or more than an initial supply and discourage the use of repeats weeks after the original has been filled
	Many patients still expect to come away from a doctor’s appointment with an antibiotic prescription, especially for a child with respiratory symptoms or middle ear infection-despite these often being self-limiting
	I believe that more public education is necessary for people to understand when antibiotics are appropriate and when they are not
Policy support to define pharmacist’s role	Pharmacists are definitely in an ideal position to be able to intervene when inappropriate antibiotic use is evident—however, the means by which the program is introduced is essential
	Pharmacists already have the knowledge and correct attitude to reduce antibiotic misuse, we just need the authority
	I genuinely think most people are unaware of what pharmacists are able to do and what we are supposed to do
Improper use of repeat prescriptions	A good start would be modifying the prescribing software to force prescribers to actually decide whether a repeat is necessary or not, rather than automatically defaulting to a repeat for every patient
	I think that antibiotic scripts should have a 2 week expiry—unless for a long-term condition. It would save repeats being saved and presented at other times …
Lower than recommended dose of antibiotics in children	Often once a week have to call doctor to adjust dose of antibiotic for children as often under dosed. Often doctors don’t tell if they need repeat or not
	Notice lower then recommended children antibiotic doses, when double check with doctors they prefer to use lower doses anyway
Impact of AMS on the business model of pharmacy	There is absolutely a need to have better remuneration for pharmacies involved in AMS programs—if the pharmacist involved is effectively performing their role, they may in fact be reducing script volume of antibiotics and thus negatively affecting the pharmacy’s takings. For instance in a pharmacist encourages a doctor to cancel a prescription for an antibiotic that is unnecessary, the pharmacy is missing out on (for example) a $10 sale. The whole process of contacting the GP, then discussing the decision with the patient may take up 15–20 min of the pharmacist’s time and ultimately the pharmacy is down $10
	We are time poor, with rapidly reducing income with health dept. and govt. who do not respect us. But still expect us to enable initiatives with little or no remuneration

## Discussion

We report the development and validation of the first questionnaire to measure the current practices and perceptions of AMS amongst Australian community pharmacists. The mixed method approach of using EFA, expert opinion and qualitative feedback to validate the survey tool was found useful in retaining important items for each section, yet reducing the size of the questionnaire to a manageable length. The three perception scales demonstrated reasonable internal validity as evident from the results of EFA and an acceptable reliability demonstrated by a Cronbach’s alpha of > 0.5 [21]. Khan et al. [7], Erku [8] and a recent study by Sarwar et al. [22] have surveyed Malaysian, Ethiopian and Pakistani community pharmacists about AMS respectively. The contexts of pharmacy practice in these countries are significantly different to those found in most Western countries, including Australia where antibiotics are available only with a valid prescription. Additionally, based on the reported results, the authors have not conducted a formal exploratory factor analysis to examine the internal structure of the questionnaire.

### Principal findings of the pilot survey

Our findings highlight that Australian pharmacists contribute to triaging common infectious presentations, determining those conditions that may require medical attention and those which are minor ailments amenable to self-care or management with over the counter medicines. This particular role of community pharmacists is not as widely appreciated in Australian settings as it has been in other countries. For example, provision of advice for minor ailments is considered a reimbursable activity in the United Kingdom [23]. We found that pharmacists were less comfortable about intervening with the choice of antibiotics or advising patients on the use of repeat prescriptions when compared to other activities such as intervening on the dose and duration of antibiotics and counselling patients regarding the adverse effects of antibiotics. Qualitative comments further provided clarification about the contextual limitations of the Australian community pharmacy practice in determining the appropriateness of antibiotics or contacting GPs for interventions related to paediatric antibiotic dosing. These findings are not surprising, as community pharmacists in Australia do not have access to a patient’s clinical and laboratory data. Additionally, unlike the UK and most of Scandinavia, community pharmacies in Australia do not operate within a healthcare network which may be limiting one-on-one interaction between GPs and pharmacists [24].

Most pharmacists in this study rated the importance of AMS highly, considering it a source of motivation and learning, potentially enhancing the public image of the profession. Likewise, pharmacists also believed that AMS programs in the community will reduce inappropriate antibiotic use and healthcare costs. Our results are consistent with the study of Burger et al. [25] where the majority of respondents indicated that AMR is a worldwide problem and pharmacists have an important role to play in tackling this problem. The perceived barriers pharmacists reported in the study included lack of access to patient’s clinical and laboratory data and lack of co-operation from the GP when the community pharmacist intervenes regarding selection of antibiotics, both of which can be inter-related. Our findings are in line with a systematic review by the National Institute of Health Research, England [26] which reported that barriers to implementing AMS include lack of resources, patients’ expectations regarding antibiotics and the influence of colleagues on the selection of antibiotics. Most of the respondents believed that educational activities targeted towards pharmacists and patients will enable them to perform AMS duties efficiently. Similarly, most of the community pharmacists suggested improved collaboration with prescribers and patients’ clinical and laboratory data would be helpful and enable them to better participate in AMS. This is consistent with the updated statement from the International Pharmaceutical Federation which stressed the importance of pharmacist and public educational initiatives in implementing AMS [27].

### Awareness of AMS and national quality initiatives

The findings of the pilot study identified a gap between AMS awareness and utilisation of available resources by community pharmacists. More efforts to engage pharmacy students, interns and pharmacists are required to develop community pharmacist’s competency in AMS. The majority of respondents were not regularly referring to the resources and activities of NPS-MW. Almost half of the respondents reported that they are not currently utilising the educational resources available to them but would definitely employ these if they get its easy access. Globally an increasing number of learning and training courses and toolkits are offered by public and private organisations, institutions and countries [28–31], some of which are free web-based online courses and others are inter-professional curriculums to increase pharmacists’ competency in AMS. There is a clear a need for such initiatives to help bridge this AMS knowledge gap for Australian community pharmacists.

### Limitations and strengths

The findings of our study should be interpreted with some caution. We only examined the views of pharmacists from one Australian state (Tasmania) and the views may not be generalizable to all Australian community pharmacists. Given the traditionally poor response rate with survey studies, we utilised a convenient sample of pharmacists drawn from a pool whose details are on file in the Division of Pharmacy, at the University of Tasmania, and this may further limit the generalisability of our findings. In contrast, a few specific strengths of the study should also be highlighted. To the best of our knowledge, this is the only study to report the development and validation of a questionnaire to measure community pharmacists’ perceptions of, and barriers to AMS in the community setting. We employed a robust process combining quantitative and qualitative data while supplementing it with expert opinion to develop and refine the questionnaire.

## Conclusion

The newly developed questionnaire to measure pharmacists’ perceptions of and barriers to, AMS in community settings demonstrated acceptable reliability and validity. Pharmacists were supportive of their involvement in the AMS, though they highlighted some important barriers limiting this involvement. A future Australia-wide study, employing the newly developed tool, will provide more data to examine the questionnaire’s reliability and validity while providing further insights into the perceptions and practices of community pharmacists regarding AMS at a national level.

## Electronic supplementary material

Below is the link to the electronic supplementary material.

Supplementary material 1 (DOCX 49 kb)
