# Supplementary material for: Perceptions and current practices of community pharmacists regarding antimicrobial stewardship in Tasmania
Source: Int J Clin Pharm. 2018 Aug 2;40(5):1380–7. doi: 10.1007/s11096-018-0701-1 (PMC6208572; doi:10.1007/s11096-018-0701-1)
Supplement: Supplementary file 1 — Supplementary material 1 (DOCX 49 kb) [file 11096_2018_701_MOESM1_ESM.docx]

**Supplementary Data**

**Appendix 1**. Exploratory factor analysis of perception items-final rotated factor solution

| **Item number**  **(n=30)** | **Item statement** | **Importance**  **1**  **Understanding** | **Importance 2**  **Motivating** | **Barriers**  **1**  **Operational** | **Barriers**  **2**  **GP Support** | **Facilitators** |
| --- | --- | --- | --- | --- | --- | --- |
| 2 | Community pharmacist can play an important role in AMS. | **-.608** | .232- | -.165 | .104 | .124 |
| 4 | AMS will reduce health care costs associated with infections. | **-.839** | .309 | .102 | -.074 | .026 |
| 5 | AMS will reduce antibiotic use in community. | **-.870** | .144 | .144 | -.006 | .179 |
| 9 | AMS will reduce inappropriate antibiotic use. | **-.611** | .038 | -.113 | .110 | -.001 |
| 6 | AMS will enhance the public image of pharmacists. | -.564 | **.780** | -.151 | -.018 | -.058 |
| 7 | AMS will enhance the job satisfaction of pharmacists. | -.416 | **.500** | -.462 | -.279 | .211 |
| 11 | I do not have the required training to participate in AMS. | .084 | -.164 | **.547** | .011 | .142 |
| 12 | I do not have enough time to participate in AMS. | .030 | -.145 | **.860** | .290 | -.155 |
| 15 | Limited access to patient’ records to review the appropriateness of antibiotic prescriptions. | -.103 | -.073 | **.867** | -.072 | .108 |
| 17 | GPs are not receptive to pharmacists intervening on the choice of antibiotic. | .213 | .352 | .024 | **.680** | -.016 |
| 18 | GPs are not receptive to pharmacists intervening on the dose of antibiotic. | .215 | .235 | .142 | **.744** | .094 |
| 19 | GPs are not receptive to pharmacists intervening on the duration of an antibiotic. | .102 | .113 | .320 | **.873** | -.167 |
| 20 | GPs are not receptive to pharmacists intervening on the dosage form of an antibiotic. | .096 | .031 | .049 | **.973** | -.178 |
| 21 | Increased provision of education activities regarding AMS | -.109 | -.049 | -.194 | -.234 | **.500** |
| 26 | Better collaboration with local GP practices. | -.108 | .034 | -.046 | -.177 | **.857** |
| 27 | Clarifications of the duties of pharmacists’ professional organisations. | .087 | -.190 | .035 | -.064 | **.509** |
| 29 | Better access to patient’s clinical and laboratory data. | .005 | -.190 | -.085 | -.152 | **.564** |
| 1 | AMS will lead to better patient outcomes. | -.470 | .114 | -.023 | -.245 | .231 |
| 3 | General practitioners are the ones who should be involved in AMS. | -.122 | .002 | .146 | .093 | .044 |
| 8 | AMS will have a minimal impact on antibiotic resistance. | .259 | .138 | .002 | .394 | -.120 |
| 10 | AMS are not practical for community pharmacy. | .288 | -.062 | .116 | .272 | -.136 |
| 13 | There aren’t any standard guidelines for community pharmacists to implement AMS programs | .093 | .170 | .230 | .317 | .047 |
| 14 | General practitioners are not welcoming of pharmacists’ involvement in AMS programs. | .265 | .126 | .218 | .396 | .200 |
| 16 | Patients do not trust pharmacist to intervene regarding an antibiotic prescription. | .318 | .296 | .134 | .464 | -.169 |
| 22 | Public awareness initiatives highlighting community pharmacists in AMS. | -.072 | .456 | -.284 | .087 | .066 |
| 23 | Computerised prompts to pharmacists while dispensing an antibiotic (e.g. checking dose, indication, etc.) | -.151 | .011 | .060 | -.076 | .180 |
| 24 | Access to guidelines for common community infections. | -.245 | .324 | .282 | -.184 | .338 |
| 25 | Provision of drug information resources. | -.142 | .247 | -.021 | -.049 | .486 |
| 28 | Monetary compensation for the time involved in AMS programs. | .018 | -.024 | .118 | -.103 | .123 |
| 30 | Targeted professional development programs in Infectious Diseases and AMS. | -.078 | .153 | -.305 | -.113 | .399 |

Loadings ≥ 0.4 are presented in bold

Extraction method: Maximum likelihood

Rotation method: Oblimin with Kaiser Normalisation

**Appendix 2.** Reliability Statistics-Item total statistics

|  | **Scale mean if item deleted** | **Scale variance if item deleted** | **Corrected item correlation** | **Cronbach’s Alpha if item deleted** |
| --- | --- | --- | --- | --- |
| **Perceived importance of AMS-Understanding of the role**  Cronbach’s Alpha .699 | | | | |
| Community pharmacist can play an important role in AMS. (n=68) | 11.721 | 4.174 | .510 | .622 |
| AMS will reduce health care costs associated with infections. (n=68) | 11.765 | 3.824 | .572 | .544 |
| AMS will reduce inappropriate antibiotic use. (n=68) | 12.397 | 3.258 | .490 | .666 |
| **Perceived importance of AMS-Motivating forces** |  |  |  | .734 |
| AMS will enhance the public image of pharmacists. (n=67) | 5.721 | 1.488 | .580 |  |
| AMS will enhance the job satisfaction of pharmacists. (n=67) | 5.676 | 1.386 | .580 |  |
| **Perceived barriers of AMS-Operational barriers**  Cronbach’s Alpha .585 | | | | |
| I do not have the required training to participate in AMS. (n=66) | 14.652 | 10.015 | .346 | .531 |
| I do not have enough time to participate in AMS. (n=64) | 14.652 | 9.092 | .419 | .469 |
| Limited access to patient record to review the appropriateness of antibiotic prescriptions. (n=65) | 12.561 | 11.327 | .425 | .489 |
| There aren’t any standard guidelines to implement AMS. (n=62) | 13.409 | 10.553 | .305 | .562 |
| **Perceived barriers of AMS-Perceived support from GPs**  Cronbach’s Alpha .890 | | | | |
| GPs are not receptive to pharmacists intervening on the choice of antibiotic. (n=63) | 11.636 | 31.712 | .609 | .909 |
| GPs are not receptive to pharmacists intervening on the dose and dosage form of antibiotic. (n=64) | 12.712 | 25.747 | .781 | .850 |
| GPs are not receptive to pharmacists intervening on the duration of antibiotic. (n=62) | 13.015 | 25.061 | .859 | .818 |
| **Perceived facilitators of AMS-General facilitators**  Cronbach’s Alpha .615 | | | | |
| Increased provision of education activities regarding AMS. (n=65) | 13.400 | 3.181 | .310 | .601 |
| Better collaboration with local GP practices. (n=65) | 13.215 | 2.890 | .597 | .459 |
| Clarifications of the duties of pharmacists’ professional organizations. (n=63) | 13.877 | 1.860 | .402 | .614 |
| Better access to patient’s clinical and laboratory data. (n=64) | 13.231 | 2.837 | .431 | .525 |
